# Effect of Iron Supplementation on the Outcome of Non-Progressive Pulmonary *Mycobacterium tuberculosis* Infection

**DOI:** 10.3390/jcm8081155

**Published:** 2019-08-02

**Authors:** Afsal Kolloli, Pooja Singh, G. Marcela Rodriguez, Selvakumar Subbian

**Affiliations:** The Public Health Research Institute Center of New Jersey Medical School, Rutgers University, Newark, NJ 07103, USA

**Keywords:** tuberculosis, latent infection, pulmonary, rabbit, *Mycobacterium tuberculosis*, iron supplementation, pathology, immune response, gene expression, Perls’ stain

## Abstract

The human response to *Mycobacterium tuberculosis* (Mtb) infection is affected by the availability of iron (Fe), which is necessary for proper immune cell function and is essential for the growth and virulence of bacteria. Increase in host Fe levels promotes Mtb growth and tuberculosis (TB) pathogenesis, while Fe-supplementation to latently infected, asymptomatic individuals is a significant risk factor for disease reactivation. However, the effect of Fe-supplementation on the host immunity during latent Mtb infection remains unclear, due partly to the paucity in availability of animal models that recapitulate key pathophysiological features seen in humans. We have demonstrated that rabbits can develop non-progressive latency similar to infected humans. In this study, using this model we have evaluated the effect of Fe-supplementation on the bacterial growth, disease pathology, and immune response. Systemic and lung Fe parameters, gene expression profile, lung bacterial burden, and disease pathology were determined in the Mtb-infected/Fe- or placebo-supplemented rabbits. Results show that Fe-supplementation to Mtb-infected rabbits did not significantly change the hematocrit and Hb levels, although it elevated total Fe in the lungs. Expression of selected host iron- and immune-response genes in the blood and lungs was perturbed in Mtb-infected/Fe-supplemented rabbits. Iron-supplementation during acute or chronic stages of Mtb infection did not significantly affect the bacterial burden or disease pathology in the lungs. Data presented in this study is of significant relevance for current public health policies on Fe-supplementation therapy given to anemic patients with latent Mtb infection.

## 1. Introduction

About a third of the world’s population is estimated to be infected with *Mycobacterium tuberculosis* (Mtb), the etiological agent of tuberculosis (TB) [1,2]. In more than 90% of Mtb-infected individuals, the immune response controls the infection, with Mtb persisting in a quiescent state [1]. These latentlyinfected (LTBI) individuals can active Mtb replication and symptomatic disease upon host immune compromising conditions [1]. Despite the role of host immunity in controlling Mtb infection, the specific components that contribute to the establishment and/or reactivation of LTBI remain poorly understood [3,4].

The human response to Mtb infection is affected by the availability of iron (Fe), which is necessary for the proper function of the immune system [5]. About a quarter of the total Fe in the body is present/stored in the hepatocytes and macrophages, while more than 60% is bound by hemoglobin, which is essential for the transfer and transport of oxygen [5]. About 5% of Fe in the body exist in proteins involved in respiration and energy metabolism. Thus, Fe homeostasis is important for normal cell growth and replication. In addition, several Fe-containing enzymes and proteins such as myeloperoxidase, NADPH oxidase, nitric oxide synthase, indole-amine 2,3 dioxygenase and lipoxygenase are directly involved in the host immune response to infection [5].

The World Health Organization (WHO) has identified Fe deficiency as the most common nutritional disorder worldwide and has developed a strategy to eradicate anemia in TB-endemic countries that includes increasing Fe intake through food fortification programs (https://www.who.int/nutrition/publications/en/ida_assessment_prevention_control.pdf). However, this program does not screen individuals for prior exposure to Mtb before starting therapy. This is a matter of concern because Fe is also essential for the growth and virulence of Mtb. Since host Fe homeostasis is tightly regulated, Mtb has to compete with the host cell to acquire Fe. As an intracellular pathogen, Mtb thrives mostly in macrophages, which contain high levels of cytoplasmic Fe. First, the host produces Fe-binding proteins, such as transferrin, ferritin and lactoferrin, in order to deprive invading pathogens of Fe and macrophages sequester these host proteins [6,7]. Second, macrophages degrade senescent erythrocytes, which release Fe-containing heme [8]. However, cytoplasmic Fe is not readily accessible to the intracellular Mtb that survive in the phagosomes. To acquire host Fe, Mtb produces Fe-chelating siderophores, which can scavenge Fe from non-heme host proteins and also facilitate the accumulation of Fe within the phagosomes of infected macrophages [8]. In infected host cells, Mtb-containing phagosomes can fuse with early endosomes, which contain transferrin, a protein that binds and transports Fe in the blood, allowing Mtb to acquire transferrin-bound Fe [9]. Increases in host Fe levels promote Mtb growth and TB pathogenesis [10,11,12]. This is evidenced by a recent study, which showed that in Fe-deficient microenvironments, such as within hypoxic granulomas, Mtb adapts to a non-replicating persistent state [13]. In addition, Esx-3, one of the type VII secretion systems of Mtb that is involved in Fe-acquisition has also been suggested to interfere with the host functions that restrict Fe-availability [14]. Moreover, Mtb mutant for *esx3* was impaired for Fe-uptake [14]. Similarly, Mtb mutant for *mbtK*, a mycobactin synthase gene involved in siderophore production, is defective for Fe-acquisition and showed attenuated growth in vitro and in infected mouse lungs [15]. These findings clearly show that Mtb utilizes several mechanisms to acquire Fe from the host and highlight the importance of Fe acquisition for the growth and virulence of Mtb. Indeed, treatment of Mtb-infected mice with Fe promoted bacterial growth in tissues and Fe-supplementation to anemic individuals has been associated with reactivation of LTBI [16,17,18]. Thus, providing Fe-supplementation therapy to asymptomatic LTBIs can be a significant risk factor for Mtb reactivation to symptomatic disease. In order to develop new and/or better public health strategies to control TB in high-burden countries, it is necessary to understand the role of host Fe homeostasis in host-pathogen interaction(s) during Mtb infection. 

Although Fe is known to promote Mtb growth, the effect of Fe-supplementation on the course of initial Mtb infections and its influence on the host immunity to infection is unclear [10]. Commonly used animal models, such as mice fail to recapitulate key pathophysiological features associated with LTBI. Previously, we have demonstrated that Mtb CDC1551-infected rabbits can develop LTBI spontaneously, as seen in humans [19]. In this study, we have used this LTBI rabbit model to investigate the effect of moderate Fe-supplementation on the immune response and outcome of pulmonary Mtb infection.

## 2. Materials and Methods

### 2.1. Bacteria and Chemicals

*Mycobacterium tuberculosis* CDC1551 (Mtb CDC1551) strain was obtained from Dr. Thomas Shinnick (Centers for Disease Control (CDC), Atlanta, GA), grown in Middlebrook 7H9 medium supplemented with 10% OADC enrichment (Difco BD, Franklin Lakes, NJ, USA), aliquoted and stored frozen at −80 °C. To prepare the inoculum for rabbit aerosol infection, stock vials were thawed and used as described previously [19]. All chemicals were purchased from Sigma (Sigma-Aldrich, St. Louis, MO, USA), unless mentioned otherwise.

### 2.2. Experimental Design

Based on our previous experience with the rabbit model of LTBI and on the literature for Fe-supplementation therapy, we set out to investigate: (a) The effect of Fe-supplementation during “acute phase” of Mtb infection (i.e., from day 1 until 8 weeks post-infection) and (b). the effect of Fe-supplementation of rabbits with a pre-established infection, i.e., starting at 8 until 16 weeks post-infection, when untreated rabbits usually develop a non-progressive “chronic phase of infection” that ultimately leads to LTBI [19]. 

### 2.3. Rabbit Infection and Treatment

Fifty-eight (*n* = 58) specific pathogen free, female New Zealand white rabbits (*Oryctolagus cuniculus*) of ~2.5 kg body weight (Covance Research Products, Denver, PA, USA) were exposed to Mtb CDC1551-containing aerosols using a “nose-only” delivery system to deliver ~3 log_10_ CFU into the lungs, as described previously [19]. At 3 hours post-exposure (T = 0), six rabbits were euthanized, lungs were harvested, and serial dilutions of the lung homogenates were plated on Middlebrook 7H11 agar media (Difco BD, Franklin Lakes, NJ, USA) to determine the number of bacterial colony-forming units (CFU). Starting at day 1 or at 8 weeks post-infection, groups of rabbits (*n* = 6 per group per time point) were randomly allocated and treated with 25 mg Fe-dextran (in 0.5 mL water) or placebo (0.5 mL sterile dextran in water) injected intra-muscularly (biceps femoris), as described previously [20]. Fe-supplementation was given 3 days a week for 8 weeks (i.e., either from day 1 until 8 weeks post-infection for acute infection, or from 8 to 16 weeks post-infection for chronic infection) (Supplementary Figure S1). This dose of Fe-dextran III (75 mg/week) is equivalent to the recommended pediatric human dose, which is non-toxic and well tolerated in rabbits [20]. The animals were given food and water ad-libitum and, weighed periodically. At T = 0 (3 h), 4, 8, 12, and 16 weeks post-infection, groups of rabbits (*n* = 6) were euthanized and organs were harvested to enumerate Mtb CFU, histology and for total host RNA isolation. About 40% (by weight) of the lung was used for preparing homogenates for Mtb CFU assays. Four animals were used as uninfected controls for Fe estimation and gene expression analysis in the blood and lung tissues. Lung tissues for RNA isolation were snap-frozen at −80 °C immediately after removal. Tissue sections for histology were fixed in neutral formalin. Blood was collected in heparinized tubes and plasma was separated by centrifugation and used in iron estimation assays. The blood pellet was used for total RNA isolation. All animal procedures were performed as per the approved procedures of the Rutgers University Institutional Animal Care and Use Committee. 

### 2.4. Histology Staining

Formalin-fixed lung portions were paraffin embedded and cut into 5 µm sections for staining with Hematoxylin–eosin (H&E) to visualize leukocytes, or Perls’ staining method for iron deposition as reported previously [21]. The stained sections were analyzed using a Nikon Microphot DXM 1200C microscope and photographed using NIS-Elements software (Nikon Instruments Inc., Melville, NY, USA). Visualization of Mtb in infected rabbit lung sections was performed by immunohistochemistry (IHC) using anti-Mtb antibody, as reported previously [22]. The IHC-stained sections were analyzed and photographed using an EVOS FL fluorescence microscope (Thermo Fisher Scientific, Pittsburg, PA, USA).

### 2.5. Measurement of Blood Parameters

Hematocrit was performed manually using heparinized capillary tubes (Fisher Scientific, Pittsburg, PA, USA), as described previously [23]. Hemoglobin (Hb) was measured by using Hemoglobin Assay Kit following the manufacturer’s instructions (Abnova, Walnut, CA, USA).

### 2.6. Measurement of Plasma and Lung Tissue Iron

Total iron-binding capacity (TIBC), total iron, and percentage transferrin saturation (%Tf) in plasma and lung homogenates of uninfected or Mtb CDC1551-infected rabbits with or without Fe-treatment was determined using a colorimetric assay (TIBC and Serum Iron Assay Kit) following the manufacturer’s instructions (BioVision, Milpitas, CA, USA). This experiment was performed in duplicate with samples from three animals per time point per condition.

### 2.7. RNA Isolation from Rabbit Lung and Blood

Total host RNA was isolated as described earlier [24]. Briefly, lung tissue and whole blood cell pellet were homogenized with 10× volume (wt/vol) of TRI reagent (Molecular Research Center, Cincinatti, OH, USA). The homogenates were extracted with 0.3 volumes (vol/vol) of BCP solution and the aqueous phase was mixed with an equal volume of 70% ethanol and passed through mini spin columns (Qiagen Inc, Germantown, MD, USA). Following on-column digestion with DNaseI, and subsequent washings, RNA was eluted with sterile water. The quality and quantity of the purified RNA was assessed in a NanoDrop instrument (NanoDrop products, Wilmington, DE, USA). 

### 2.8. Quantitative Real-Time PCR Analysis (qPCR)

Total RNA was reverse transcribed into cDNA using the AffinityScript QPCR cDNA Synthesis Kit following instructions of the manufacturer (Agilent Technologies Inc., Santa Clara, CA). Quantitative PCR (qPCR) experiments were performed on a Stratagene Mx3005p machine (Agilent Technologies, Inc. Santa Clara, CA, USA) with cDNA and site-specific oligonucleotide primers of target genes, using Brilliant III Ultra-Fast SYBR® Green QPCR Master Mix, according to the product instructions (Agilent Technologies Inc., Santa Clara, CA, USA). No SYBR and no cDNA control samples were included in one of the triplicate assays for each experimental time point. Housekeeping gene *GAPDH* was included to normalize the levels of expression of test genes. Threshold cycle value (C_t_) was determined using MxPro4000 software and fold-change in gene expression was calculated from the formula 2^–ΔΔCt^, where ΔC_t_ is the difference in C_t_ between the test gene and *GAPDH* and ΔΔC_t_ is the difference between test and control conditions. Each experiment was repeated at least three times with cDNA from 3 to 6 animals per experimental time point per group. Supplementary Table S1 lists the description of tested genes and primer sequences used in qPCR experiments. 

### 2.9. Statistical Analysis

Statistical analysis was performed on data by one-way ANOVA followed by Tukey’s multiple test comparison using GraphPad Prism-5 (GraphPad Software, La Jolla, CA, USA) and the mean ± standard deviation values were plotted as graphs. For all the experimental data, *p* ≤ 0.05 was considered statistically significant.

## 3. Results

### 3.1. Iron Parameters in Fe-Supplemented Rabbits during Acute or Chronic Mtb Infection 

The effect of Fe-supplementation on the hematocrit and hemoglobin (Hb) content of rabbits during Mtb-infection was measured at 4 and 8 weeks (acute) or 12 and 16 weeks (chronic) post- infection/treatment.

An average hematocrit level of 42% and Hb content of ~14 g/L was observed at all time-points (Supplementary Figure S2), and no significant difference in either the hematocrit or Hb levels was observed in Mtb-infected rabbits with Fe- or placebo supplementation. 

### 3.2. Systemic and Lung Fe Levels in Mtb-Infected Rabbits with or without Fe-Supplementation

The total Fe binding capacity (TIBC), which is an indirect measure of free Fe, total Fe, and the percent of Fe-saturated transferrin (%Tf) were measured in rabbit plasma and lung homogenate.

**Figure 1:**
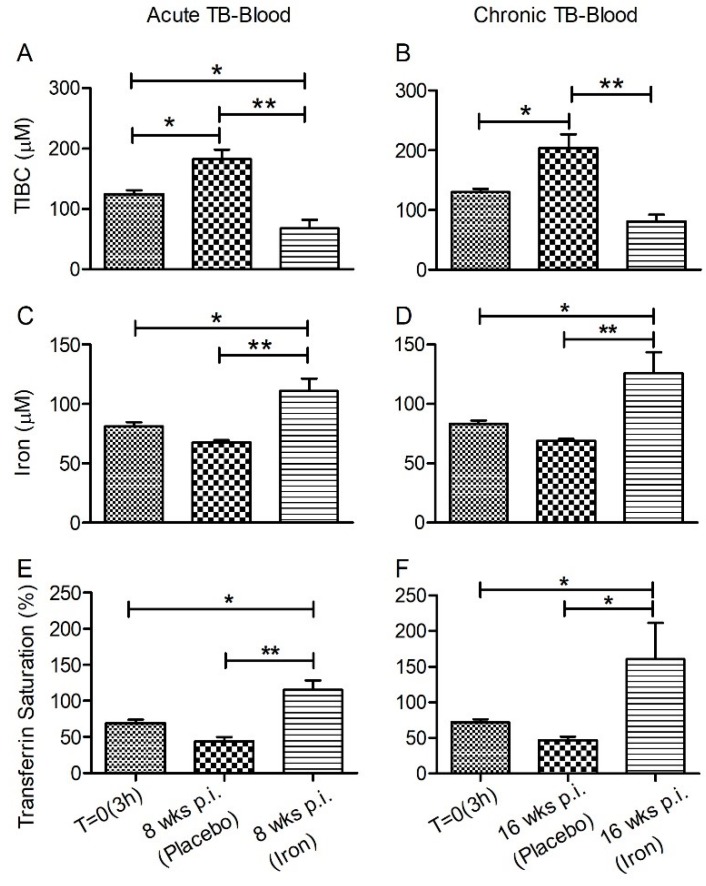
Effect of Fe-supplementation on plasma iron parameters of rabbits at 8 weeks (**A**,**C**,**E**) or 16 weeks (**B**,**D**,**F**) post-Mtb infection. Total iron binding capacity (TIBC) (top), total iron (middle), and percent transferrin saturation (bottom) were determined in plasma samples of Mtb-infected and placebo- or Fe-treated rabbits at the conclusion of treatment (i.e., 8 weeks or 16 weeks post-infection). Data was analyzed by one-way Anova with Tukey’s multiple comparison test. Values plotted are mean +/– sd with *n* = 4 per group per time point. * *p* < 0.05; ** *p* < 0.01.

**Figure 2:**
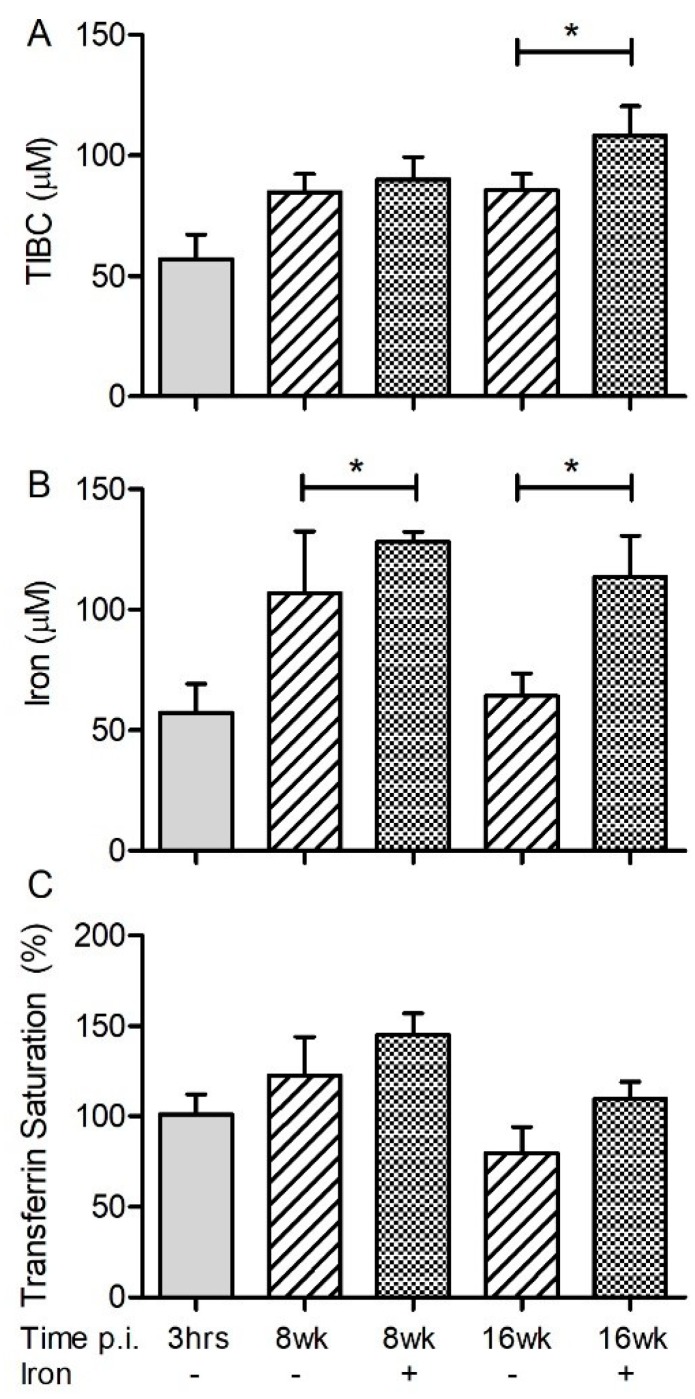
Effect of Fe-supplementation on the lung iron parameters of rabbits at 8 and 16 weeks post infection. TIBC (top; **A**), total iron (middle; **B**) and percent transferrin saturation (bottom; **C**) were determined in the homogenates of Mtb-infected and placebo (-) or Fe (+) treated rabbits. Data was analyzed by one-way Anova with Tukey’s multiple comparison test. Values plotted are mean +/– sd with *n* = 4 per group per time point. * *p* < 0.05.

In the plasma, Mtb-infected rabbits with Fe-supplementation had significantly reduced TIBC, accompanied by elevated total Fe and %Tf saturation, compared to placebo-treated animals at both acute and chronic stages of infection (Figure 1A–C). In the lungs, no significant differences in TIBC and %Tf saturation levels were observed between animals, irrespective of their Fe-supplementation status during the acute phase of infection (Figure 2A,C). However, a higher Fe level was observed in the lungs of Mtb-infected/Fe-supplemented animals, compared to placebo-treated animals during acute (8 weeks) and chronic (16 weeks) post infection (Figure 2B). Fe-supplementation during chronic Mtb infection significantly increased the lung TIBC only at 16 weeks, compared to the placebo-treated rabbits (Figure 2A). Similarly, the Fe-supplemented rabbits also had a significantly higher Fe level in the lungs at 4 and 12 weeks post infection, compared to the placebo-supplemented animals (Supplementary Figure S3). 

### 3.3. Fe-Supplementation Does Not Affect Systemic Host Iron-Responsive Gene Expression during Mtb Infection

The effect of Fe-supplementation on the systemic expression of host iron-responsive genes, including *HFE1*, *HFE2* (*HJV*), *HFE3* (*TFR2*), *HAMP*, *SLC40A1* (*FPN1*), *SLC11A2* (*NRAMP2*), *HMOX1*, *FTH1*, *LCN2*, *BMP6,* and *NFE2L2* (*NRF2*) was determined in Mtb-infected rabbit blood and lungs. 

These genes are selected for their role in systemic Fe homeostasis as well as host response to Fe in the system (Supplementary Table S2). Among these genes, *HFE1* and *HFE2* interact with the transferrin receptor (TFR) and transferrin to modulate Fe transportation and availability. Transferrin also controls expression of hepcidin (HAMP), the central regulator of systemic Fe [25,26]. Moreover, HFE2 is a co-receptor for BMP, which drives HAMP expression through the SMAD signaling pathway [27]. The Fe exporter, FPN, mediates export of Fe recycled in macrophages from erythrocytes and Fe absorbed from the diet by enterocytes. HAMP binds to FPN and induces its internationalization and subsequent degradation, thereby reducing Fe release from macrophages and enterocytes [28]. FTH1 is a subunit of ferritin, an Fe-storage protein [29], and HMOX is an enzyme involved in heme catabolism, both of which are regulated by host Fe status [30]. NRF2, a regulator of the antioxidant response, also regulates FTH1 and FPN to maintain cellular Fe homeostasis [31]. LCN2 is an immune protein that sequesters Fe-loaded siderophores and blocks bacterial Fe acquisition from infected host cells [32].

**Figure 3:**
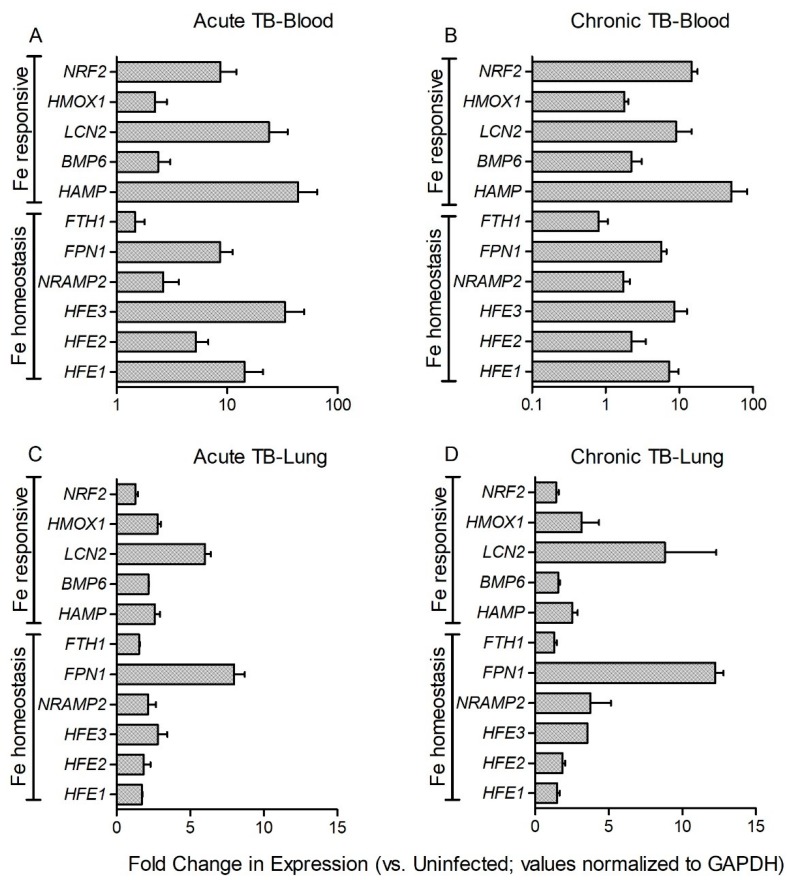
Expression of iron-responsive genes in *Mycobacterium tuberculosis* (Mtb)-infected rabbits at 4- and 12-weeks post infection. Data shown are expression of target genes in the blood (**A**,**B**) or lung (**C**,**D**) during acute (8 weeks; **A**,**C**) or chronic (16 weeks; **B**,**D**) stages of infection. The gene expression levels in Mtb-infected animals was calibrated with the corresponding levels in uninfected rabbits. Host house-keeping gene (*GAPDH*) expression was used to normalize the level of target gene expression. Data was analyzed by one-way Anova with Tukey’s multiple comparison test. Values plotted are mean +/– sd with *n* = 4 per group per time point. All tested genes were statistically significant in Figure 3A–D.

**Figure 4:**
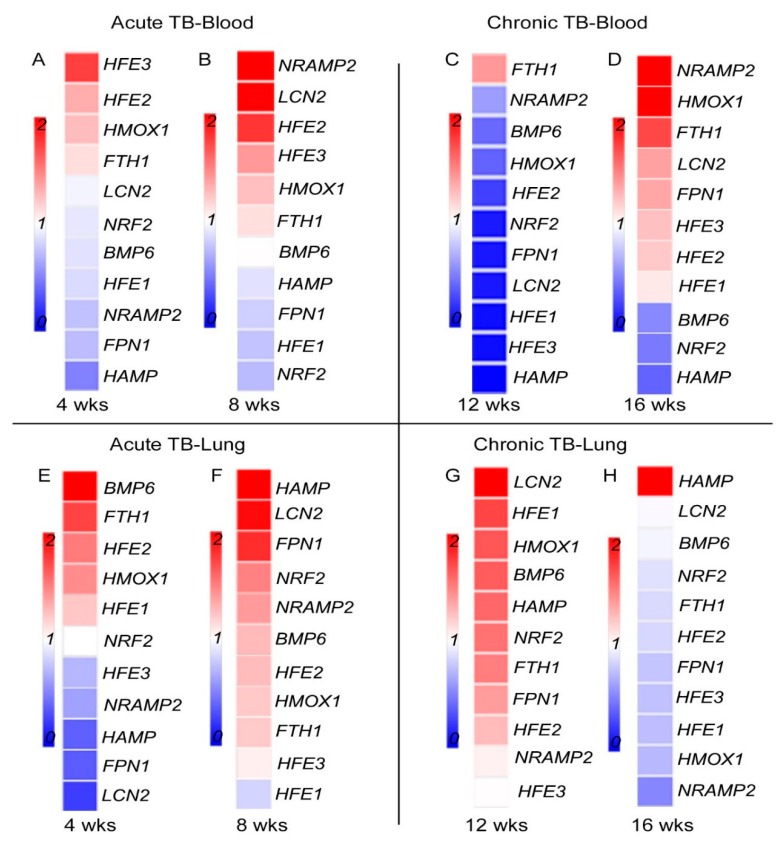
Effect of Fe-supplementation on the expression of host iron-responsive genes in Mtb-infected rabbits. Heat map of selected immune response gene expression in the blood (**A**–**D**) or lung (**E**–**H**) during acute (4 and 8 weeks; **A**,**B**,**E**,**F**) or chronic (12 and 16 weeks; **C**,**D**,**G**,**H**) stages of infection in rabbits treated with Fe. The data from Fe-supplemented animals was calibrated with the corresponding levels in placebo-treated animals. Host house-keeping gene (*GAPDH)* expression was used to normalize target gene expression. Red color indicates up-regulation and blue color indicates down-regulation. The gradient in color indicates the trend towards up- or down-regulation.

Consistent with activation of nutritional immunity, expression of all the tested Fe-responsive genes was significantly up-regulated in the blood of Mtb-infected rabbits at 8 weeks (acute) (Figure 3A) and 16 weeks (chronic) (Figure 3B) post infection, compared to the uninfected animals. Similar trend in the expression of these genes was noted in rabbit blood at 4 (acute) and 12 (chronic) weeks post infection (Supplementary Figure S4). However, no significant differential expression was observed in the blood between placebo- and Fe- supplemented Mtb-infected rabbits at any of the tested time points (Figure 4A–D). These results suggest that Mtb infection induces the expression of host Fe-responsive genes systemically and that Fe-supplementation does not lead to further up-regulation of these genes. 

### 3.4. Fe-Supplementation Perturbs Expression of Host Iron-Responsive Genes in the Lungs of Mtb-Infected Rabbits 

The effect of Fe-supplementation on the expression of *HFE1*, *HFE2*, *HFE3* (*TFR2)*, *BMP6*, *HAMP*, *FPN1*, *NRAMP2*, *HMOX1*, *FTH1*, *NRF2,* and *LCN2* was assessed in rabbit lung homogenates. Expression of all the tested genes was significantly up-regulated in the lungs of Mtb-infected/placebo treated rabbits at 8 (acute) and 16 (chronic) weeks post-infection, compared to uninfected animals (Figure 3C,D). A similar trend in the expression of these genes was noted in Mtb-infected rabbit lungs at 4 (acute) and 12 (chronic) weeks post infection (Supplementary Figure S4). Fe-supplementation resulted in a significant down-regulation of *HFE3* (*TFR2*), *HAMP, FPN1,* and *LCN2* while *BMP6* and *FTH1* were significantly up-regulated, compared to the placebo group, at 4 weeks post-infection (Figure 4E). At 8 weeks post-infection, expression of *BMP6*, *HAMP*, *FPN1, FTH1, NRF2,* and *LCN2* was significantly up-regulated, while *HFE1* was significantly down-regulated by Fe-supplementation, compared to the placebo group (Figure 4F). Expression of *BMP6* and *FTH1* were up-regulated by Fe-supplementation at both, 4 and 8 weeks post-infection.

Among the tested Fe-responsive genes, expression of *HFE1*, *BMP6,* and *HMOX1* at 12 weeks, and *HAMP* at 16 weeks post-infection, was significantly up-regulated. At these later time points, expression of *HFE1*, *HFE3 (TFR2),* and *FPN1* was significantly down-regulated by Fe-treatment, compared to the placebo group (Figure 4G,H). Taken together, these results indicate that Mtb infection induces the expression of host Fe-responsive genes in rabbit lungs and that a subset of these genes were differentially regulated by Fe-supplementation during acute and chronic stages of infection. 

### 3.5. Fe-Supplementation Affects Systemic Expression of Host Immune Response Genes in Mtb-Infected Rabbits

**Figure 5:**
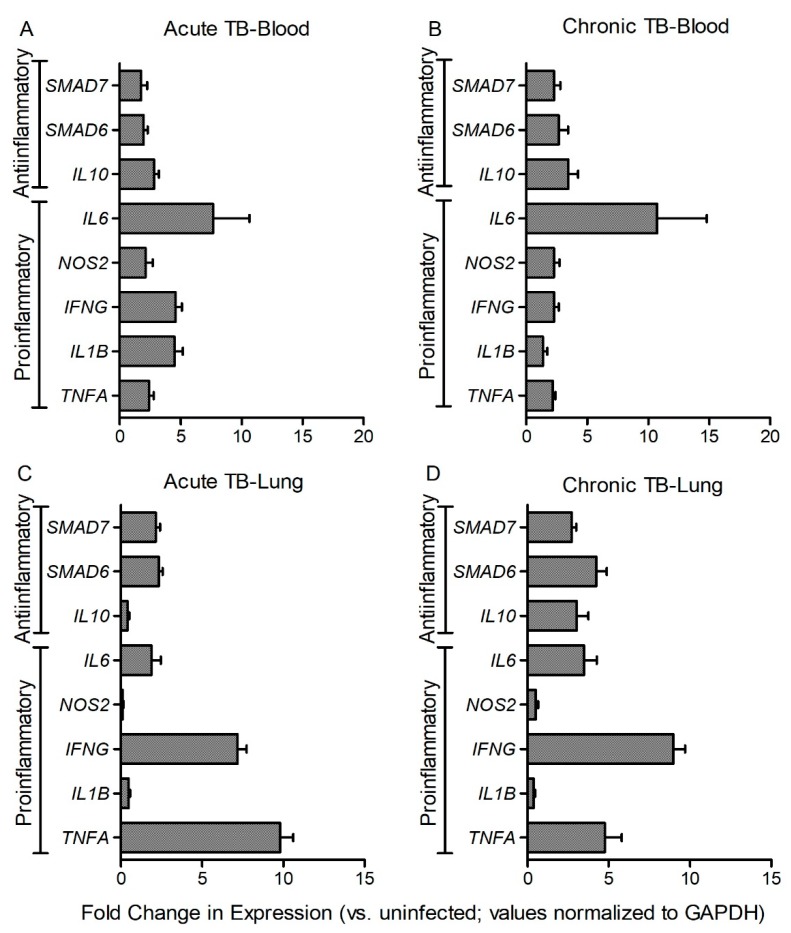
Expression of host pro- and anti-inflammatory response genes in Mtb-infected rabbits at 8 and 16 weeks post infection. Data shown are expression of target genes in the blood (**A**,**B**) or lung (**C**,**D**) during acute (8 weeks; **A**,**C**) or chronic (16 weeks; **B**,**D**) stages of infection. The gene expression levels in Mtb-infected animals was calibrated with the corresponding levels in uninfected rabbits. Host house-keeping gene (*GAPDH*) expression was used to normalize the level of target gene expression. Data was analyzed by one-way Anova with Tukey’s multiple comparison test. Values plotted are mean +/– sd with *n* = 4 per group per time point. All tested genes were statistically significant (*p* < 0.05) in Figure 5A–D.

**Figure 6:**
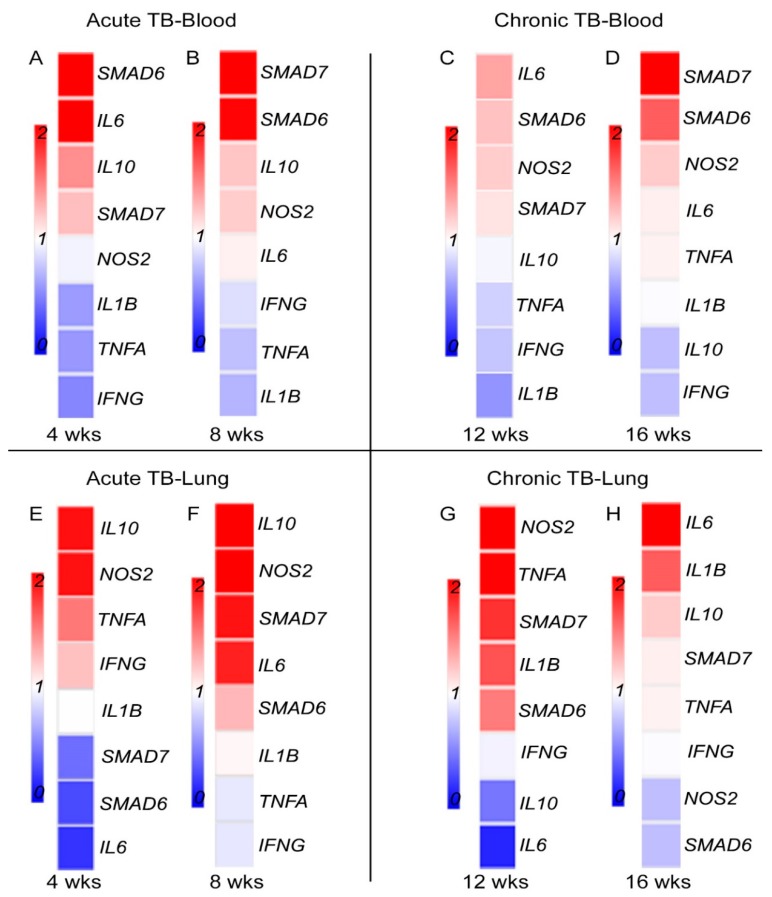
Effect of Fe-supplementation on the expression of host immune response genes in Mtb-infected rabbits. Heat map of selected immune response gene expression in the blood (**A**–**D**) or lung (**E**–**H**) during acute (4 and 8 weeks; **A**,**B**,**E**,**F**) or chronic (12 and 16 weeks; **C**,**D**,**G**,**H**) stages of infection in rabbits treated with Fe. The data from Fe-supplemented animals was calibrated with the corresponding levels in placebo-treated animals. Host house-keeping gene (*GAPDH)* expression was used to normalize target gene expression. Red color indicates up-regulation and blue color indicates down-regulation. The gradient in color indicates the trend towards up- or down-regulation

To determine the association between host iron- and immune-response gene expression at systemic level, we measured the expression of proinflammatory (*IFNG*, *TNFA*, *IL1B, NOS2,* and *IL6*) and antiinflammatory (*IL10, SMAD6,* and *SMAD7*) genes in the blood of Mtb-infected and placebo- or Fe-supplemented rabbits. While proinflammatory cytokines are involved in mounting a protective immunity, anti-inflammatory molecules are involved in wound healing and dampening of inflammatory response [10,19]. SMAD6 and SMAD7 are involved in HAMP expression through BMP signaling [23] (Supplementary Table S2). In the blood and lung homogenates of Mtb-infected rabbits, the level of expression of all tested genes was significantly up-regulated, at 8 (acute) and 16 (chronic) weeks post infection (Figure 5A–D). Similar trend in the expression of these genes was noted in the blood and lungs at 4 (acute) and 12 (chronic) weeks post infection (Supplementary Figure S5). In Mtb-infected/Fe-supplemented rabbits, expression of *IFNG*, *TNFA,* and *IL1B* was significantly down-regulated, whereas expression of *IL6* and *SMAD6* was up-regulated, compared to placebo-treated animals, at 4 weeks post-infection (Figure 6A). At 8 weeks post-infection, expression of *SMAD6* and *SMAD7* was significantly up-regulated in the Mtb-infected/Fe-supplemented animals, compared to placebo-treated rabbits (Figure 6B). Although expression of all the tested genes was up-regulated in the blood at 12 and 16 weeks post-infection, Fe-supplementation did not significantly alter the expression of any of the tested genes at these time points (Figure 6C,D). 

These results suggest that expression of host immune-response genes was significantly up-regulated systemically during acute and chronic stages of Mtb infection. Fe-supplementation during acute, and not chronic stages of infection, reduced the expression of a subset of proinflammatory genes while up-regulating antiinflammatory genes at the systemic level.

### 3.6. Fe-Supplementation Perturbs Expression of Host Immune Response Genes in the Lungs of Mtb-Infected Rabbits

Expression of *IFNG*, *TNFA*, *IL1B, NOS2, IL6, IL10*, *NOS2*, *SMAD6,* and *SMAD7* was measured at 4 and 8 (acute) or 12 and 16 (chronic) weeks post infection, using total RNA isolated from lung homogenates of rabbits with placebo- or Fe-supplementation (Figure 6E–H). Compared to uninfected, the level of expression of all tested genes were significantly up-regulated in the lungs during acute and chronic stages of Mtb infection, irrespective of treatment status. In Mtb-infected/Fe- supplemented rabbits, expression of *SMAD6* and *SMAD7* was significantly down-regulated at 4 weeks, compared to the placebo-treated animals (Figure 6E). At 8 weeks post-infection, expression of *IL10, NOS2,* and *SMAD7* was significantly up-regulated in the Fe-supplemented animals (Figure 6F). 

Although the tested host immune response genes were highly up-regulated in the lungs at 12 and 16 weeks post-infection (Figure 5 D and Supplementary Figure S5), no significant difference in the expression level was observed for any of the tested genes between Fe-supplemented and placebo-treated rabbit lungs at these time points (Figure 6G,H). 

These results suggest that Mtb infection up-regulates the expression of the tested host immune response genes at the site of infection, and a subset of these genes were differentially regulated by Fe-supplementation during acute infection.

### 3.7. Fe-Supplementation Did Not Affect the Lung Bacillary Load in Mtb-Infected Rabbits 

**Figure 7:**
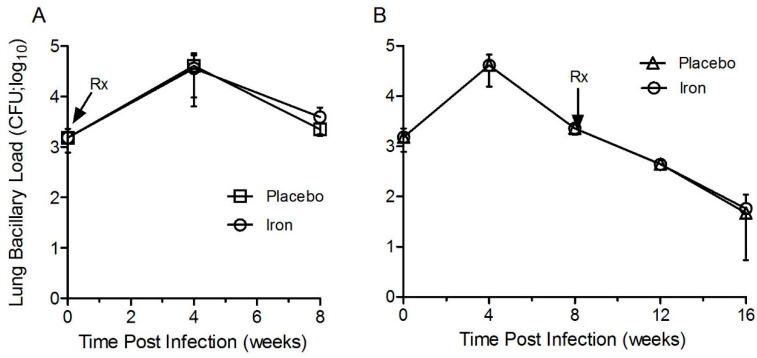
Effect of Fe-supplementation on the lung bacillary load of Mtb-infected rabbits. Number of bacterial colony-forming units (CFU) was determined in the lungs of rabbits supplemented with Fe or placebo during acute (**A**) or chronic (**B**) stages of Mtb infection. Data was analyzed by one-way Anova with Tukey’s multiple comparison test. Values plotted are mean +/– sd with *n* = 5 per group per time point. Rx denotes treatment start time (day-1 or 8 weeks post-infection). The treatment was continued for 8 weeks in both acute and chronic infection groups.

To test whether Fe-supplementation affects the progression of initial infection to active disease (acute phase), Mtb CFU was determined at 4 and 8 weeks in the lung homogenates of rabbits with/without Fe-supplementation (Figure 7A). In Mtb-infected/placebo-treated animals, the lung bacterial CFU increased significantly, reaching a peak load at 4 weeks (3log_10_ at T = 0 to 4.5 log_10_ CFU at 4 weeks). A gradual reduction to 3.5 log_10_ CFU was seen at 8 weeks post-infection. Compared to the placebo group, Fe-supplementation from day 1 onwards until 8 weeks post-infection, did not significantly affect the bacterial CFU at both 4 and 8 weeks post-infection (Figure 7A). At these time points, no significant difference in body weight was seen between Mtb-infected/Fe-supplemented and placebo groups (Supplementary Figure S6).

To test whether Fe-supplementation affects the pulmonary bacterial burden during the chronic stage of infection, groups of Mtb-infected rabbits were supplemented with Fe or placebo for 8 weeks, starting at 8-week post-infection (until 16-week post-infection) (Figure 7B). In the placebo-treated animals, the lung bacillary load increased significantly with a peak burden of ~4.5 log_10_ CFU at four weeks, followed by a gradual decrease in CFU until 16 weeks post-infection, when the mean bacterial CFU was ~1.5 log_10_. Fe-supplementation of Mtb-infected rabbits from 8–16 weeks post-infection did not significantly affect the net cultivable bacillary load in the lungs, compared to the placebo group. (Figure 7B). No significant difference in body weight was seen between Mtb-infected/Fe-supplemented and placebo-treated rabbits at these time points (Supplementary Figure S6). 

These results suggest that Fe-supplementation during acute or chronic stages of Mtb infection did not significantly affect the bacterial burden in the lungs of infected rabbits.

### 3.8. Fe-Supplementation Did Not Affect Disease Pathology in Rabbit Lungs Infected with Mtb

**Figure 8:**
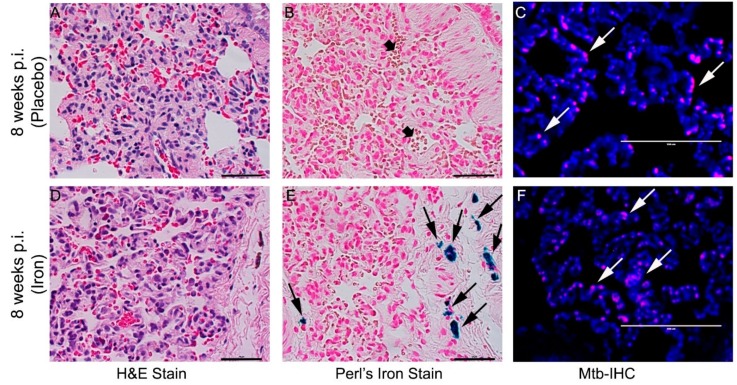
Effect of Fe-supplementation on rabbit lung pathology at 8 weeks post Mtb infection. Histopathology of rabbit lungs infected with Mtb CDC1551 at 8 weeks post infection with *(***A**–**C***)* or without *(***D**–**F***)* Fe-supplementation showing disease pathology (H&E stain; **A**,**D**), iron deposition (Perls’ iron stain; **B**,**E**) and Mtb (by immunohistochemistry; **C**,**F**). Dark arrows in (**E**) show cellular iron deposition (blue color). White arrows in (**C**,**F**) show Mtb (purple color). The scale bar for all the images is 50 µm. Sections were photographed at 400× (**A**,**B**,**D**,**E***)* or 600× (**C**,**F**) of original magnification.

To determine the effect of Fe-supplementation on TB immunopathology, we performed histologic staining of lung, liver, and spleen tissues with H&E, Perls’ Fe-staining, or AFB staining to visualize immune cells, Fe deposition, or Mtb, respectively (Figure 8).

Analysis of the H&E-stained lung sections of Mtb-infected/placebo-treated rabbits at 4 and 8 weeks showed both histopathologic and disease manifestations, as described previously (Figure 8 and Supplementary Figure S7) [19]. No clearly visible, macroscopic sub-pleural granuloma was seen in any of the infected rabbits at these time points. However, microscopic granulomas with increased immune cell infiltration, including neutrophils and monocytes, were prominent at 4 and 8 weeks post-infection, although no central necrosis or caseation was noticed in the granulomas. The granulomas in Mtb-infected/Fe-supplemented rabbit lungs at 4 and 8 weeks were very similar in size, cellular architecture, and maturation state compared to Mtb-infected/placebo-treated animals (Figure 8 and Supplementary Figure S7). 

Fe deposition in lung granulomas was evaluated by Perls’ staining method. Fe deposition was observed in the histiocytes of lung lesions only in Fe-supplemented and not in placebo-treated rabbits at 4 (Supplementary Figure S7) and 8 weeks post-infection/treatment (Figure 8B,E). At these time points, the presence of Mtb was observed by immunohistochemistry using anti-Mtb antibody in the lung lesions of both Fe-supplemented and placebo-treated, rabbits (Figure 8C, F and Supplementary Figure S7). 

**Figure 9:**
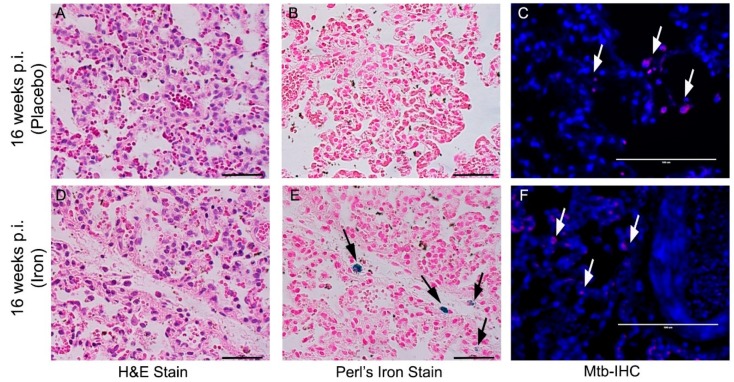
Effect of Fe-supplementation on rabbit lung pathology at 16-weeks post Mtb infection. Histopathology of rabbit lungs infected with Mtb CDC1551 at 16 weeks post infection with (**A**–**C**) or without (**D**–**F**) Fe-supplementation showing disease pathology (H&E stain; **A**,**D**), iron deposition (Perls’ iron stain; **B**,**E**) and Mtb (by immunohistochemistry; **C**,**F**). Dark arrows in (**E**) show cellular iron deposition (blue color). White arrows in (**C**,**F**) show Mtb (purple color). The scale bar for all the images is 50 µm. Sections were photographed at 400× (**A**,**B**,**D**,**E***)* or 600× (**C**,**F**) of original magnification.

Histologic analysis of the H&E-stained lung sections of Mtb-infected/placebo-treated rabbits during chronic stages of infection (i.e, 12 and 16 weeks post infection) showed similar histopathology and disease manifestations, as described previously (Figure 9 and Supplementary Figure S8) [19]. No macroscopic sub-pleural lesions were observed in Mtb-infected rabbit lungs at these time points. However, a small number of microscopic cellular aggregations, reminiscent of resorbing lesions, with fewer immune cells present, were prominent at 12 weeks post-infection (Supplementary Figure S8). By 16 weeks, the lungs had a small number of cellular aggregates within the otherwise normal-looking lung parenchyma, consistent with our previous report (Figure 9) [19]. The lung lesions of Fe-supplemented rabbits at 12 and 16 weeks post-infection were very similar in size and cellular infiltration to the placebo-treated animals at the same time points (Figure 9 and Supplementary Figure S8). Perls’ staining method revealed Fe deposition in the lung cells at 12 and 16 weeks post-infection/treatment only in the Fe-supplemented, and not in placebo-treated, rabbits (Figure 9B,E and Supplementary Figure S8). At these time points, Mtb was present in the lung sections of Fe-supplemented and placebo-treated animals (Figure 9C,F and Supplementary Figure S8).

These observations show that although elevated Fe was present in the lungs of Mtb-infected/Fe-supplemented rabbits at both acute and chronic stages of infection, the course of infection and disease pathology were not significantly altered by the supplementation.

## 4. Discussion

Here, we have determined the effect of Fe-supplementation on the outcome of non-progressive, pulmonary Mtb CDC1551 infection in a rabbit model. We show that Fe-supplementation alters the level of TIBC, total Fe, and %Tf saturation, and expression of host iron- and immune-response genes at the systemic and local levels during Mtb infection. Histologic examination of rabbit lungs revealed accumulation of Fe in Mtb-infected/Fe-supplemented rabbits without significant change in lung bacterial burden or disease pathology, compared to placebo-treated rabbits. 

We observed significant up-regulation of Fe-responsive host gene expression in the lungs and in the blood of rabbits during acute and chronic stages of Mtb infection. Our findings are consistent with, and supported by previous reports in vitro and in mice, guinea pig, and non-human primate models of pulmonary TB [33,34,35,36]. These studies have shown that during Mtb infection, Fe-responsive host genes, such as *FPN1*, *FTH1*, *LCN2*, *HMOX1,* and *HAMP1*, were differentially regulated as a host-protective response against infecting bacteria. Some of these genes, such as *HAMP1* and *BMP6,* were also reported to be differentially expressed between lungs and liver of mycobacteria-infected mice [37]. Although gene expression in blood was not reported in these studies, we observed a differential expression pattern of host iron-responsive genes between blood and lung cells in Mtb-infected rabbits. We also observed differential TIBC, total Fe, and %Tf saturation levels between blood and lung homogenate of Mtb-infected rabbits. The association between Fe parameters and host iron-responsive gene expression in blood and lungs, and the relevance of this association to Mtb pathogenesis, requires further investigation.

We observed that Fe-supplementation did not significantly alter the expression of host iron-responsive genes that were up-regulated by Mtb infection in rabbit blood and lungs. The up-regulation of these genes, including *HAMP1*, *BMP6,* and *FTH1* in the lungs of Fe-supplemented rabbits, is likely part of the host’s response to increased Fe accumulation. This is supported by histologic examination, which revealed accumulation of Fe in the lungs of Mtb-infected/Fe-supplemented rabbits. The induction of *HAMP* suggests down-regulation of Fe absorption and retention in macrophages; increased *HAMP* expression through induction of BMP pathway has been reported in human immune cells exposed to high Fe and in a mouse model of Mtb infection supplemented with Fe [38,39]. Taken together, these results suggest that: (a) Mtb infection induces the expression of host iron-responsive genes in rabbits—a subset of these genes were differentially regulated by Fe-supplementation and/or by infection stage (acute versus chronic); and (b), host iron-responsive genes are differently regulated in blood and lungs of Mtb-infected rabbits.

During the course of Mtb infection, expression levels of many host immune-response genes were up-regulated both systemically (blood) and locally (lungs) in untreated rabbits. Most of these genes, including *TNFA*, *IL1B*, *IFNG,* and *IL10,* were up-regulated during acute phase of infection and their expression gradually declined, following immunological control the infection, at later stages in *Mtb*-infected rabbits. These findings are consistent with our earlier reports on humans, and in various animal models, that showed elevated expression of pro- and anti- inflammatory genes during Mtb infection [19,40]. Fe-supplementation of Mtb-infected rabbits reduced the expression of these host immune response genes systemically, particularly during acute phase of infection, but not in the lungs. However, expression of *IL6*, *IL10, SMAD6,* and *SMAD7* were up-regulated both in the lungs and in blood of Mtb-infected/Fe-supplemented rabbits. This is consistent with previous findings showing up-regulation of antiinflammatory cytokine genes, such as *IL10*, and down-regulation of the proinflammatory Th1 response during Fe-supplementation [10]. Similarly, reduction of *TNFA*, *IFNG,* and *IL1B* expression, with concomitant induction of *IL10* expression, has been reported previously in Fe-treated *M. bovis* BCG-infected mice, in Mtb-infected murine cell line J774, and in human mononuclear phagocytes [37,41,42]. In addition, up-regulation of IL-6, as a consequence of an acute response due to proinflammatory stimuli, has been shown to induce HAMP production in THP-1 cells, human macrophages, and in a murine model [38,43,44]. Taken together, these findings are consistent with our observations, and highlight the influence of Fe in the host immune response to Mtb infection. 

We observed that perturbation of either the systemic or the local expression of host immune- and iron-responsive genes during acute and chronic stages of Mtb infection did not affect the outcome of infection. The organ bacillary loads, as well as disease pathology, were similar between Fe-supplemented and placebo-treated Mtb-infected rabbits. This finding is consistent with the data from recent studies in murine models of pulmonary TB with Fe-supplementation which showed that neither Fe-supplementation nor Fe deficiency significantly altered TB susceptibility and that Mtb load in infected organs was similar between Fe-supplemented/Fe-deficient and control mice [15,34,37]. One possibility for this observation is that although elevated Fe accumulation was noted in Mtb-infected/Fe-supplemented hosts, the Fe may not be available to infecting bacteria to enhance their growth and/or to cause exacerbated disease [45]. Consistently, %Tf saturation levels were significantly elevated in Fe-supplemented/Mtb-infected rabbits. Transferrin can sequester excess Fe in the host, thus restricting its availability to Mtb. In contrast, another study showed increased Mtb growth upon Fe overload in a mouse model of pulmonary infection [46]. Similarly, exacerbated disease burden was reported in a guinea pig model of Mtb infection with Fe overload [47]. One reason for the discrepancy between these reports and our study on Mtb infection and Fe overload is that most of the animal and human studies on Fe-supplementation were conducted in an active TB condition, marked with progressive Mtb infection/disease, exacerbated inflammation, and bacillary loads in affected tissues [46,47]. In contrast, the rabbit model of LTBI used in this study develops non-progressive infection, with an initial protracted period of bacterial growth and a protracted granulomatous response, followed by a gradual reduction in bacillary load and disease pathology, both of which are efficiently controlled over time as the rabbits develop latency [19]. Therefore, the impact of Fe-supplementation is likely affected by the model system, the nature of infecting Mtb strain, and the subsequent host response. 

The relationship between host Fe status and the outcome of Mtb infection is complex and not yet fully understood [10,11,34,37,38]. Patients with active TB, and some animal models of Mtb infection, have been shown to develop anemia, resulting from both Fe deficiency and/or chronic inflammation [48,49,50]. However, the association between host susceptibility to mycobacterial infection and host Fe status remains unclear [34,48,49,50,51,52,53,54,55,56,57]. A recent study showed that almost half of all patients with active pulmonary TB had anemia due to Fe deficiency and/or inflammation, at the time of diagnosis, and were recommended Fe supplementation as part of their therapy [57]. However, the association between anemia and LTBI remains unclear, although anemia is considered as a major risk factor for disease reactivation among these individuals [55,56,57]. Moreover, it has been reported that Fe-supplementation is beneficial to the host in situations where Fe deficiency co-exists with active disease showing exacerbated disease pathology [58]. In other conditions, where disease-associated inflammation predominates without concurrent Fe deficiency, the overall benefit of Fe-supplementation on the host is either negligible or insignificant [49]. In support of this notion, a study reported that Fe-supplementation to 131 pulmonary TB patients did not influence Mtb growth or disease severity, compared to the placebo group [49]. Similarly, reports on household contacts and LTBI individuals showed no significant correlation between Fe parameters and progression of disease [56]. Thus, in chronic, non-progressive Mtb infections, such as our study reported here, in which there was no exacerbated inflammation and associated disease pathology, Fe-supplementation may not have a significant influence on the outcome of Mtb infection. 

In conclusion, our findings did not show a causal role for Fe supplementation and reactivation of LTBI in terms of bacterial burden and tissue pathology, although we observed changes in host gene expression associated with Fe-homeostasis and host immunity in Fe-supplemented animals,. However, results from this study, which links moderate Fe-supplementation with the host immunity during latent Mtb infection, should facilitate future investigations to determine the components of immune system that modulate Fe-dependent responses and to identify Mtb factors that perturb host Fe homeostasis. 

## Supplementary Materials

The following are available online at https://www.mdpi.com/2077-0383/8/8/1155/s1, Table S1: Description of primers used in this study; Table S2: Description of Fe and immune genes tested in this study; Figure S1: Scheme for rabbit Mtb infection and Fe-treatment; Figure S2: Effect of Fe-treatment on the hematocrit (top) and hemoglobin (bottom) content of rabbits with acute (A) or chronic (B) Mtb infection; Figure S3: Effect of Fe-supplementation on the lung iron parameters of rabbits at 4 and 12 weeks post infection; Figure S4: Expression of iron-responsive genes in Mtb-infected rabbits at 4 and 12 weeks post infection; Figure S5: Expression of host pro- and anti-inflammatory response genes in Mtb-infected rabbits at 4 and 12 weeks post infection; Figure S6: Body weight of Mtb-infected rabbits with or without Fe-treatment; Figure S7: Effect of Fe supplementation on rabbit lung pathology at 4 weeks post Mtb infection; Figure S8: Effect of Fe supplementation on rabbit lung pathology at 12 weeks post Mtb infection.
