# Supplementary material for: Effect of Iron Supplementation on the Outcome of Non-Progressive Pulmonary Mycobacterium tuberculosis Infection
Source: J Clin Med. 2019 Aug 2;8(8):1155. doi: 10.3390/jcm8081155 (PMC6722820; doi:10.3390/jcm8081155)

SUPPLEMENTARY MATERIAL

Supplementary Table 1. Description of primers used in this study

Supplementary Table 2. Description of Fe and immune genes tested in this study

Supplementary Figure 1. Scheme for rabbit Mtb infection and Fe-treatment

Supplementary Figure 2. Effect of Fe-treatment on the hematocrit (top) and hemoglobin (bottom) content of rabbits with acute (A) or chronic (B) Mtb-infection

Supplementary Figure 3. Effect of Fe-supplementation on the lung iron parameters of rabbits at 4 and 12 weeks post infection. TIBC (top; A), total iron (middle; B) and percent transferrin saturation (bottom; C) were determined in the homogenates of Mtb-infected and placebo- or Fe-treated rabbits. Data was analyzed by one-way Anova with Tukey's multiple comparison test. Values plotted are mean +/- sd with n=4 per group per time point. *$p<0.05$.

Supplementary Figure 4. Expression of iron-responsive genes in Mtb-infected rabbits at 4 and 12 weeks post infection. Data shown are expression of target genes in the blood (A, B) or lung (C, D) during acute (4 weeks; A, C) or chronic (12 weeks; B, D) stages of infection. The gene expression levels in Mtb-infected animals was calibrated with the corresponding levels in uninfected rabbits. Host house-keeping gene (*GAPDH*) expression was used to normalize the level of target gene expression. Data was analyzed by one-way Anova with Tukey's multiple comparison test. Values plotted are mean +/- sd with n=4 per group per time point. All tested genes were statistically significant in Figures 3A, B, C, D.

Supplementary Figure 5. Expression of host pro- and anti-inflammatory response genes in Mtb-infected rabbits at 4 and 12 weeks post infection. Data shown are expression of target genes in the blood (A, B) or lung (C, D) during acute (4 weeks; A, C) or chronic (12 weeks; B, D) stages of infection. The gene expression levels in Mtb-infected animals was calibrated with the corresponding levels in uninfected rabbits. Host house-keeping gene (*GAPDH*) expression was used to normalize the level of target gene expression. Data was analyzed by one-way Anova with Tukey's multiple comparison test. Values plotted are mean +/- sd with n=4 per group per time point. All tested genes were statistically significant in Figures 5A, B, C, D.

Supplementary Figure 6. Body weight of Mtb-infected rabbits with or without Fe-treatment.

Supplementary Figure 7. Effect of Fe supplementation on rabbit lung pathology at 4 weeks post Mtb infection. Histopathology of rabbit lungs infected with Mtb CDC1551 at 4 weeks post infection with *(A-C)* or without *(D-F)* Fe-supplementation showing disease pathology (H&E stain; A, D), iron deposition (Perls' iron stain; B, *E*) and Mtb (by immunohistochemistry; C, F). Dark arrows in E show cellular iron deposition (blue color). White arrows in C, F show Mtb (purple color). The scale bar for all the images is 50 µm. Sections were photographed at 400X (A, B, D, E*)* or 600X (C, F) magnification.

Supplementary Figure 8. Effect of Fe supplementation on rabbit lung pathology at 12 weeks post Mtb infection. Histopathology of rabbit lungs infected with Mtb CDC1551 at 12 weeks post infection with (A-C) or without (D-F) Fe-supplementation showing disease pathology (H&E stain; A, D), iron deposition (Perls' iron stain; B, E) and Mtb (by immunohistochemistry; C, F). Dark arrows in E show cellular iron deposition (blue color). White arrows in C, F show Mtb (purple color). The scale bar for all the images is 50 µm. Sections were photographed at 400X (A, B, D, E) or 600X (C, F) magnification.

Supplementary Table 1. Description of primers used in this study.

Sr. No.	Target Gene	Forward Primer 5'-3'	Reverse Primer 5'-3'
1	*BMP6*	TGGACGCACACAAGCTAGG	GGTTGAAGGAAGGGAAGCCA
2	*FPN1*	GCTCTACGCCTCCTATGTCTAC	CGTGAGACTGGTGGAGGAAG
3	*FTH1*	CCATGTGAATGCCAGCGTG	GCCTCCTGTGCCCAAGATAG
4	*HAMP*	CGTGGGAGCTGTCATCATG	GGCTTCTCGAACTTCCTGCT
5	*HFE1*	TACGTGGGGAGATCGGATGT	GCTACGACCAGGCCATAGAC
6	*HFE2*	TAGACCCGACAGCAGGACAG	TCAGGCCAGTGAGAACAAGG
7	*HFE3*	TAAGTGACTCGGAGCTGGGA	AGTGGGTTAAGTGTGGCTGG
8	*HMOX1*	GGTGGCAGGACTGGATCATC	CGTGGTTGGTTGCGTTCATG
9	*LCN2*	CTTGCTCTCAGGGATCTCGG	TCCCAGAGGTAGGAGGTCAC
10	*NRAMP2*	CTCTCTCCACAGCCACCTTC	TTCCAAACCAGTCACGGAGC
11	*NRF2*	CCCATCGACCAGTGCATTGA	GCCTCTGTGTCTCTTTGTGC
12	*IFNG*	GGTCCAGCGTAAAGCAGTAA	GAAACAGCGTCTGACTCCTT
13	*IL1B*	TGTTGTCTGGCACGTATGAG	GCCACAGGTATCTTGTCGTT
14	*IL6*	ACTGGCGGAAGTCAATCTGC	CCTGAACTTGGCCTGAAGGT
15	*IL10*	AACCACAGTCCAGCCATCAG	TGTAGACGCCTTCCTCTTGC
16	*NOS2*	AGAGACGCACAGGCAGAGGT	GCAGGCACACGCAATGATGG
17	*SMAD6*	CGGCAGCTCTTTGGGAATTT	AGGAAGGAAGGAGAGGGAGA
18	*SMAD7*	CTCACGCACTCGGTGCTCAA	GATCCGGCCACCTGAACACT
19	*TNFA*	CTGAGTGACGAGCCTCTAGC	TTCATGCCGTTGGCCAGCAG
20	*GAPDH*	GGCGTGAACCACGAGAAGTA	TCCACAATGCCGAAGTGGTC

Supplementary Table-2: Details of host genes reported in this study

Gene symbol	Description	Function	Reference cited
HFE1	Homeostatic iron regulator-1	Regulates Fe absorption	# 21, 22, 23
HFE2	Hemochromatosis type-2	Fe absorption; co-receptor to BMP	# 21, 22, 23
HFE3	Transferrin receptor-2	Fe absorption	# 21, 22, 23
BMP6	Bone morphogenic protein-6	Driver of HAMP expression	# 23
HAMP	Hepcidin antimicrobial peptide	Maintenance of iron homeostasis	# 24
FPN1	Ferroportin-1	Fe export	# 24, 25
NRAMP2	Solute carrier family 11 member 2	Fe absorption	
HMOX1	Hemeoxygenase-1	Heme catabolism	# 26
FTH1	Ferritin heavy chain-1	Subunit of ferritin, an iron storage protein	# 25, 26
NRF2	Nuclear factor, erythroid 2 like	Fe-responsive transcriptional regulator of antioxidant response	# 27
LCN2	Lipocalin-2	Sequesters Fe-loaded siderophores	# 28
IFNG	Interferon gamma	Th1 cytokine	# 6, 15, 33, 36-40
TNFA	Tumor necrosis factor alpha	Proinflammatory cytokine	# 6, 15, 33, 36-40
IL1B	Interleukin-1 beta	Inflammatory cytokine	# 6, 15, 33, 36-40
IL6	Interleukin-6	Inflammatory cytokine	# 6, 15, 33, 36-40
IL10	Interleukin-10	Anti-inflammatory cytokine	# 6, 15, 33, 36-40
NOS2	Inducible nitric oxide synthase-2	Antimicrobial response	# 6, 15, 33, 36-40
SMAD6	SMAD family member 6	Involved in HAMP expression through BMP	# 23
SMAD7	SMAD family member 7	Involved in HAMP expression through BMP	# 23

Supplementary Figure-1: Scheme for rabbit Mtb infection and treatment

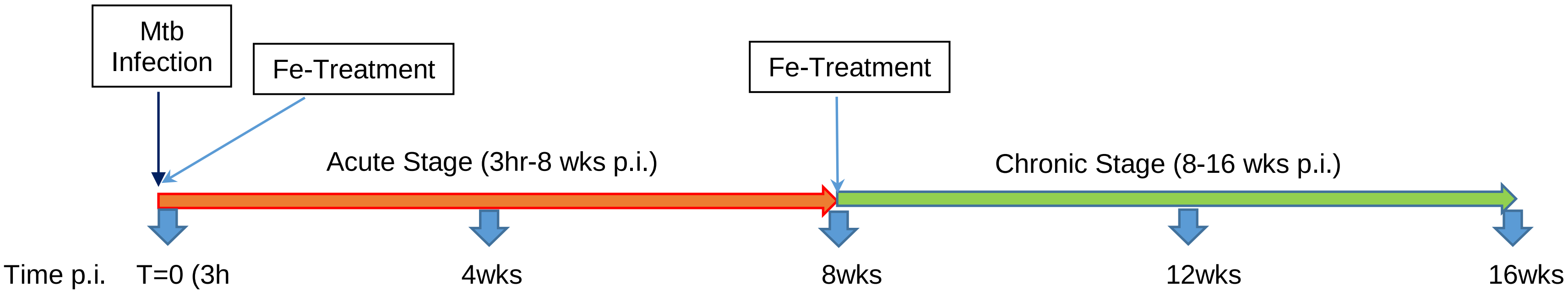

SUPPLEMENTARY FIGURE-2

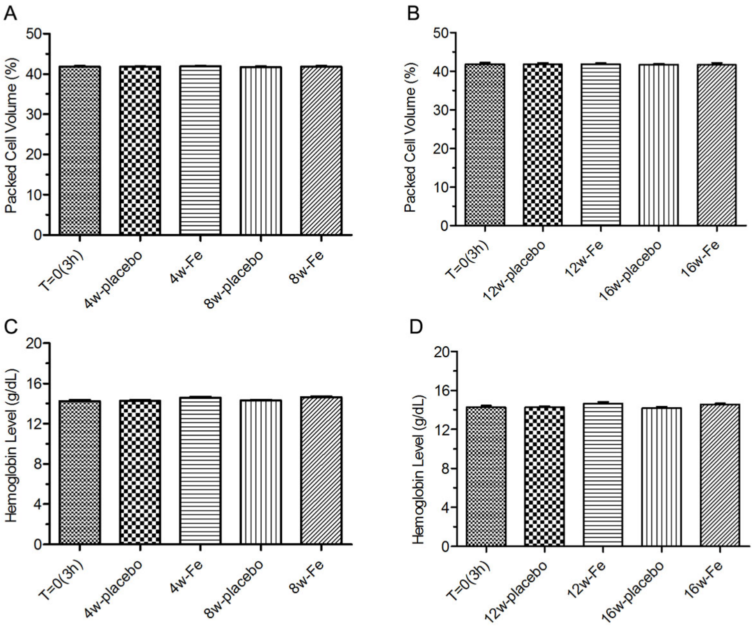

SUPPLEMENTARY FIGURE-3

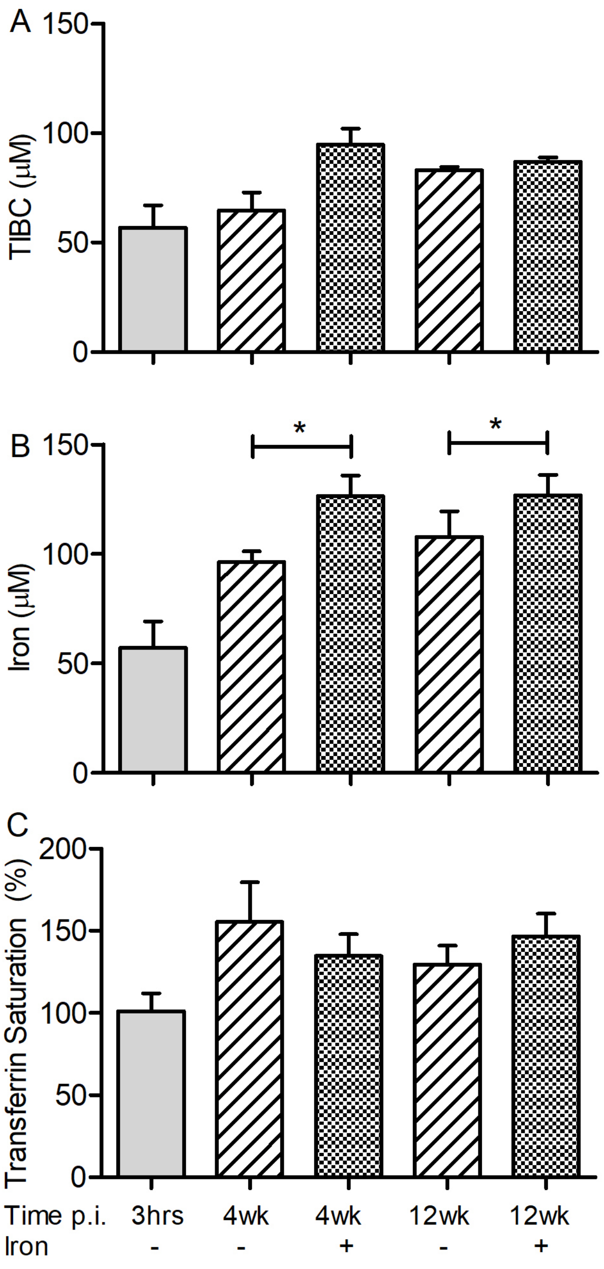

SUPPLEMENTARY FIGURE-4

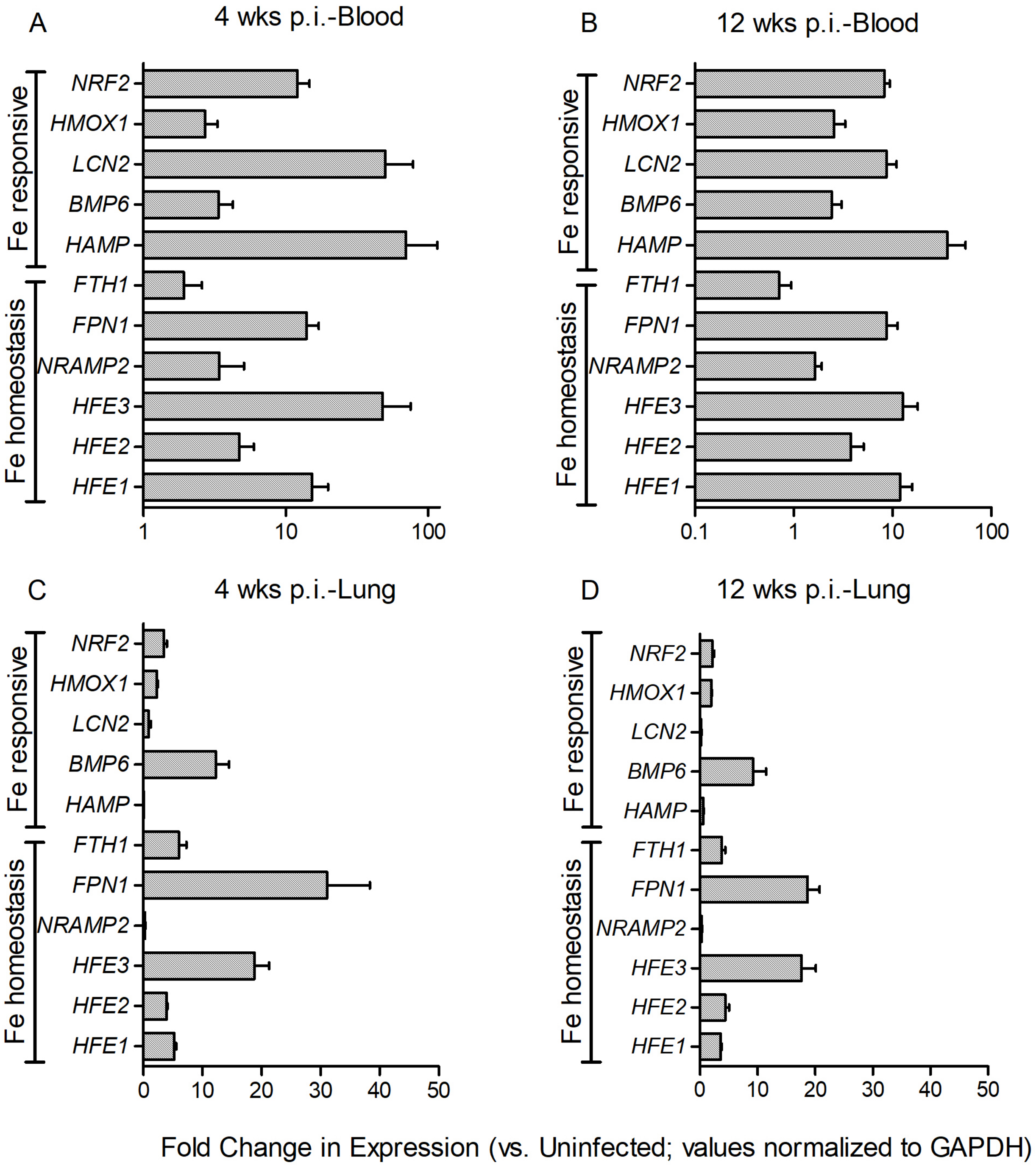

SUPPLEMENTARY FIGURE-5

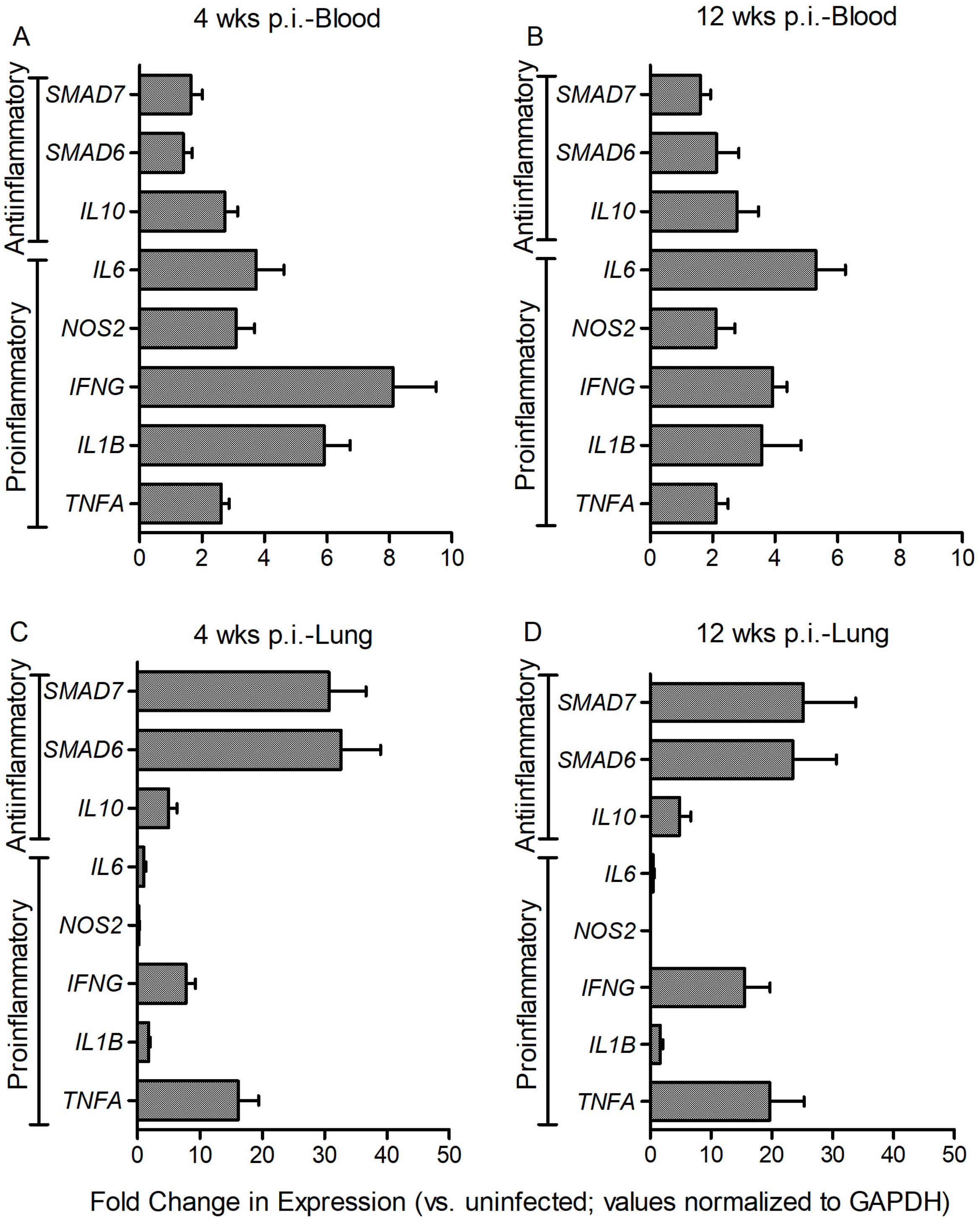

SUPPLEMENTARY FIGURE-6

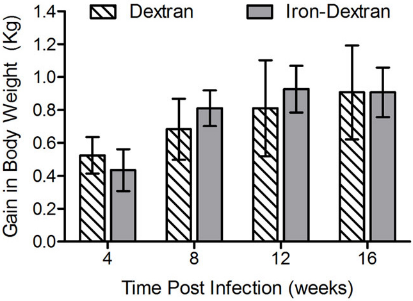

SUPPLEMENTARY FIGURE-7

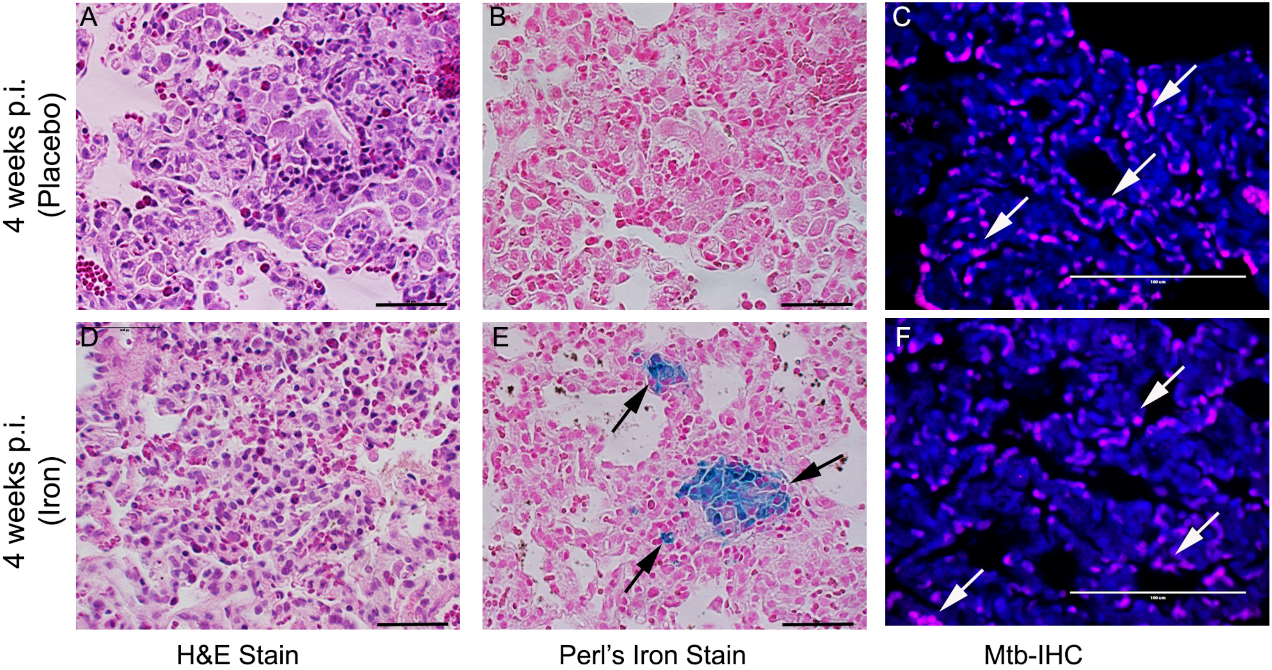

SUPPLEMENTARY FIGURE-8

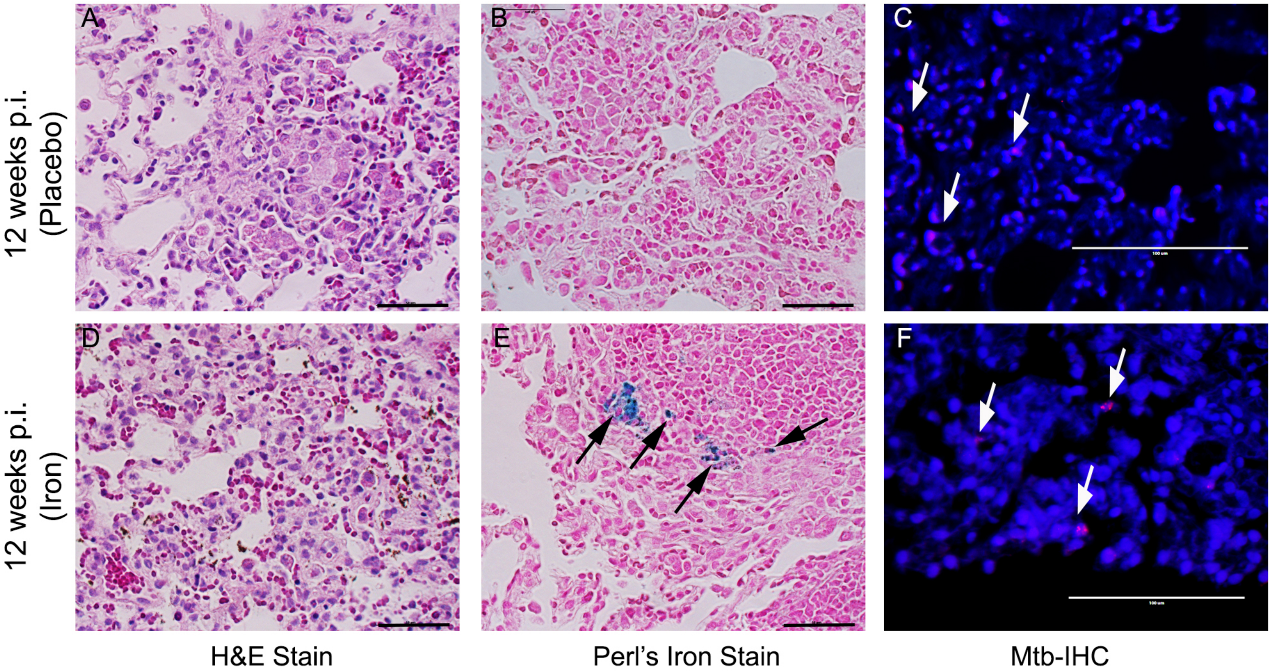

Supplement: Supplementary file 1 [file jcm-08-01155-s001.pdf]
